# Editorial: Biomarkers of Brain Damage – A Complex Challenge With Great Potential

**DOI:** 10.3389/fneur.2021.664445

**Published:** 2021-03-18

**Authors:** Olli Tenovuo, Damir Janigro, Jean-Charles Sanchez, Johan Undén

**Affiliations:** ^1^University of Turku, Turku, Finland; ^2^Turku University Hospital, Turku, Finland; ^3^Case Western Reserve University, Cleveland, OH, United States; ^4^Université de Genève, Geneva, Switzerland; ^5^Department of Anesthesiology and Intensive Care, Hallands Hospital Halmstad, Lund University, Lund, Sweden

**Keywords:** brain, injury, biomarker, proteomics, diagnostics

The brain has remained as the only organ where blood- or other fluid-based biomarkers have been practically lacking from the commonly used clinical diagnostic tools. There are several reasons for this, such as the complexity of the brain as an organ, the presence of a blood-brain barrier (BBB), extremely small concentrations of many brain-derived proteins in blood, and the difficulty of using cerebrospinal fluid for routine diagnostics. The development of biochemical analytics has enabled measuring of very small amounts of various substances, which has led to a new and promising era for brain biomarker diagnostics.

Despite the vast number of published studies, few biomarkers have thus far entered the clinic for a number of reasons. First, many target molecules are brain-enriched, and not specific for the brain or CNS. Thus, there is a need to perform control studies in several potential patient groups. Second, to what extent the biomarker reflects brain tissue damage, and to what extent the biomarker level depends on the integrity of the BBB and/or the glymphatic function, is a challenging question to clarify. Third, each biomarker has a specific kinetic profile, some appearing rapidly and some slowly depending on the clinical condition, and each with a different half-life possibly related to kidney function. Thus, it may be challenging to interpret what a single level means, giving only a narrow window for the often complex and dynamic pathophysiological events. Fourth, the fairly demanding detection technologies are prone to technical errors, thus results have to be validated in independent laboratories before broader acceptance. Fifth, collecting sufficient numbers of samples from well-characterized subjects with appropriate control groups is a significant effort, often requiring multicenter collaboration.

Despite the aforementioned challenges, there is no question that the forthcoming years and decades will see a revolution in the diagnostics of brain disorders. Biomarkers have potential uses that other methodologies cannot replace. They may give information on injuries in different cell types and separate cellular compartments, such as axonal or synaptic injury. They may be used to monitor treatment responses rapidly. They can give – when used in validated panels – a comprehensive picture about the brain state non-invasively. Not least, they may be used for point-of-care rapid diagnostics, thus opening entirely new possibilities for the clinicians.

This special issue has aimed to collect papers that advance the field. The paper by Janigro et al. reports how to assess BBB permeability and how the BBB influences brain biomarker measurement in peripheral biofluids. These kinds of studies are of utmost importance for the understanding of clinical samples. The paper by Smirl et al. also studied the neurovascular unit, as other articles focused on traumatic brain injury (TBI), by examining the alterations of the neurovascular unit after soccer headings. Four of the papers (Hossain et al.; Lagerstedt et al.; Kahouadji et al.; Posti et al.) report the value of different promising biomarkers in assessing acute TBI, and how they relate to imaging findings and outcome. A fifth paper by Huebschmann et al. reports how the levels of a much-studied glial fibrillary acidic protein (GFAP) differ in serum and plasma of older adults. The paper by Guedes et al. extends TBI-related studies beyond proteomics in reporting the use of extracellular vesicles and microRNAs as TBI biomarkers. The paper by Kawata et al. reports how some of the most promising TBI protein biomarkers associate with imaging markers of axonal injury in subjects with repeated head impacts. Although the concept “biomarker” is commonly associated with measurement of biomolecules, in a wider sense all biological measurements are biomarkers; the paper by Haider et al. reports how concussion alters responses of the parasympathetic nervous system. Finally, the paper by Quiroz-Baez et al. discusses the use of extracellular vesicles as biomarkers of neurodegenerative conditions.

We hope that this special issue stimulates further efforts in the field – there is still much to be done. Understanding how different physiological and pathophysiological phenomena affect various biomarkers, and how different biomarkers behave under multiple clinical conditions and their stages will still require intensive research. By all likelihood, the vast brain complexity can rarely be assessed using a single biomarker; we thus assume that the use of various biomarker panels and their interpretation using artificial intelligence approaches will constitute a major change in clinical neurosciences, possibly already during this decade.

## Author Contributions

OT wrote the draft. All authors revised and approved the final text.

## Conflict of Interest

The authors declare that the research was conducted in the absence of any commercial or financial relationships that could be construed as a potential conflict of interest.

